# Atrial Functional Tricuspid Regurgitation (AFTR) Is Associated with Better Outcome After Tricuspid Transcatheter Edge-to-Edge Repair (T-TEER) Compared to Ventricular FTR (VFTR)

**DOI:** 10.3390/jcm14030794

**Published:** 2025-01-25

**Authors:** Jinny Karin Scheffler, Jan-Philipp Ott, Mona Landes, Dominik Felbel, Matthias Gröger, Mirjam Kessler, Johannes Mörike, Marvin Krohn-Grimberghe, Leonhard Moritz Schneider, Wolfgang Rottbauer, Michael Paukovitsch

**Affiliations:** Department of Cardiology, Ulm University Heart Center, Albert-Einstein-Allee 23, 89081 Ulm, Germany

**Keywords:** edge-to-edge repair, atrial functional tricuspid regurgitation

## Abstract

**Background:** Transcatheter tricuspid edge-to-edge repair (T-TEER) is widely used to treat atrial (AFTR) and ventricular (VFTR) functional tricuspid regurgitation (FTR). **Methods:** The outcome of 136 patients treated with T-TEER for severe AFTR or VFTR was analyzed using a composite endpoint of all-cause death and rehospitalization for decompensated heart failure. AFTR was defined as TR in the context of left ventricular ejection fraction ≥50%, right ventricular fractional area change (RVFAC) ≥ 35% and sPAP ≤ 50 mmHg. **Results:** Patients with VFTR (N = 109) and AFTR (N = 27, 19.9%) were both elderly (82.0 {IQR: 74.5–84.5} vs. 82.0 {IQR: 75.0–84.0} years, *p* = 0.98) and had similar interventional risk according to the EuroScore II (6.1 {4.0–9.8} vs. 4.7 {3.6–9.6} %, *p* = 0.3). Atrial fibrillation was equally frequent in both groups (89.9 vs. 88.9%, *p* = 0.88). AFTR patients were significantly more often female (56.0 vs. 77.8%, *p* = 0.04) and had lower NT-proBNP (3600.0 {1706.0–6302.0} vs. 1988.0 {1034.8–3723.3} pg/mL, *p* < 0.01). While RVFAC (29.5 ± 8.6 vs. 42.1 ± 4.3%, *p* < 0.01) and LVEF (48.5 ± 12.3 vs. 58.6 ± 8.0%, *p* < 0.01) were expectedly lower in patients with VFTR, right atrial dilation (RA volume: 126.7 ± 56.5 vs. 127.6 ± 74.2 mL, *p* = 0.99) was similar. Successful T-TEER with TR reduction ≥ 2 degrees (96.3 vs. 92.6%, *p* = 0.34) was observed in both groups, and residual TR ≤ II was equally frequent (94.5 vs. 96.3%, *p* = 1.0). The incidence of the 1-year composite endpoint was significantly higher (34.3 vs. 12.0%) in patients with VFTR (log-rank *p* = 0.02). AFTR was inversely associated with the composite endpoint (HR: 0.21, 95% CI: 0.06–0.7, *p* < 0.01) in multivariate Cox regression. **Conclusions:** Despite equally effective TR reduction through T-TEER, a better outcome was observed in patients with AFTR.

## 1. Introduction

Transcatheter tricuspid edge-to-edge repair (T-TEER) has evolved to a widely adapted treatment option for severe tricuspid regurgitation [1,2]. Especially in these multimorbid patients [2,3,4,5], the assessment of pathophysiology and etiology seems more and more crucial to optimize patient selection and treatment options. More than 90% of tricuspid regurgitation (TR) is caused by secondary or functional TR (FTR) [6]. Atrial functional TR (AFTR) can be described as a distinct entity of isolated TR secondary to right atrial and tricuspid annular remodeling, in the absence of ventricular dysfunction or leaflet tethering [7]. The etiology of TR seems to have a strong prognostic impact, leading to growing interest in understanding AFTR. Patients with AFTR present with lower rates of mortality and heart failure hospitalizations compared to patients with non-atrial TR, if treated conservatively [5,8,9,10]. Data are still scarce [8,11,12] regarding the impact of TR etiology in patients undergoing T-TEER.

Moreover, an official or uniform definition of AFTR is missing so far, especially in guidelines [6,13]. Thus, recent publications used different classifications based on clinical data or echocardiographic parameters, including, e.g., atrial fibrillation, systolic pulmonary artery pressure (sPAP), left ventricular function (LVEF) and different parameters for RV function [8,11]. A recent expert opinion document and state-of-the-art review [7] published by members of the Tricuspid Valve Academic Research Council (TVARC) [14] suggested a set of criteria to differentiate AFTR from VFTR. Based on recommendations outlined in this document [7], we compared outcomes of patients with AFTR and VFTR after T-TEER.

## 2. Methods

### 2.1. Study Population and Procedural Details

This is a retrospective, monocenter study including 136 consecutive patients treated with T-TEER for severe (III+), symptomatic (≥II New York Heart Association {NYHA} class) FTR despite guideline-directed medical therapy (GDMT) between January 2022 and December 2023 at the Ulm University Heart Center. These patients were part of the TRIC-Ulm registry, the local T-TEER registry. All patients provided written informed consent for data collection and telephone follow-up. This study was approved by the local ethics committee (Ulm University Ethics committee, local approval number: 142/20, date of approval 9 September 2020).

All patients were evaluated by the interdisciplinary heart team, and T-TEER was recommended based on the heart team’s consensus. Patients with degenerative changes of the TV (severe leaflet degeneration) were classified as having primary or degenerative TR (DTR) and were excluded from this study. Furthermore, we excluded patients with TR induced by cardiac implantable electronic devices (CIED-induced TR). Patients with a CIED and TR in whom the CIED was not main cause of TR were not excluded from this study.

No further exclusion criteria existed.

T-TEER was performed under general anesthesia with transesophageal echocardiography (TEE) and fluoroscopy guidance. For catheter access, a venous access via the groin was established with ultrasound-guided vessel puncture. Both transgastric and mid-esophageal as well as lower esophageal views were used for device implantation. A suture-based closing system (Proglide, Abott Vascular, Redwood City, CA, USA) was used for vessel closure. The choice and number of devices used for the procedure was within the discretion of the interventionalist. The PASCAL Ace (Edwards Lifesciences, Irvine, CA, USA) as well as the Triclip (Abott, Chicago, IL, USA) systems were available.

### 2.2. Definition of AFTR and VFTR

The definition of AFTR used was based on a recently published expert recommendation and state-of-the-art-review [7]. AFTR was defined as left ventricular ejection fraction ≥ 50%, right ventricular fractional area change (RVFAC) ≥ 35% and sPAP ≤ 50 mmHg. An sPAP cut-off of 50 mmHg was used, as suggested by two previously published studies [10,11], to account for ventricular dysfunction secondary to severe pulmonary hypertension.

### 2.3. Echocardiography

Patients received structured transthoracic echocardiography (TTE) upon admission to the hospital using an X5-1 probe on an EpiQ Ultrasound machine (Philipps, Andover, MA, USA) and followed standards outlined in reference [13]: LVEF was determined using the biplane Simpson method in the apical 4- and 2-chamber views. Right ventricular fractional area change (RVFAC) was calculated from an RV focused view by determining the RV end-diastolic and endystolic area. SPAP was determined by measuring maximum tricuspid regurgitation and adding an estimation of central venous pressure (CVP). CVP estimation followed recommendations outlined in the ESC guideline for pulmonary hypertension [15] (inferior vena cava diameter < 2.1 cm and >50% collapse on inspiration: 3 mmHg; IVC > 2.1 and >50% IVC collapse on inspiration: 8 mmHg; IVC > 2.1 cm and <50% collapse: 15 mmHg).

Tricuspid regurgitation was graded using a 5-grade grading scheme [16] including qualitative and quantitative parameters such as 2D effective regurgitant orifice area (EROA) or 3D EROA, biplane vena contracta or 3D vena contracta and regurgitant volume as well as hepatic venous flow.

TEE was performed using an X8-2 probe with 2D and 3D imaging (depending on image quality) on the same type of echo machine.

### 2.4. Patient Follow-Up

Follow-ups were conducted either by out-patient visit or structured telephone interview, which were performed after 30 days, 1 year and yearly thereafter.

### 2.5. Study Endpoints

For the outcome analysis, a composite endpoint of all-cause death and rehospitalization for decompensated heart failure was used. The endpoint was analyzed separately for 1-year outcomes as well as for the overall outcome (using the latest available follow-up).

One-year follow-up was available for 94.1% of patients. Those patients who did not complete 1-year follow-up (lost to follow-up) were included in the analysis with the longest follow-up available.

### 2.6. Statistical Analysis

For the statistical analysis, patients were grouped according to AFTR or VFTR. For continuous variables, distribution was analyzed using histograms and Q-Q plots. Normally distributed variables are shown as mean ± standard deviation and were analyzed using the t-test. Non-normally distributed variables are shown as median and interquartile range (IQR in brackets) and were analyzed using the Mann–Whitney Test.

Categorical variables are shown as absolute numbers and frequencies, and the Chi-square test or Fisher’s exact test were used for variable comparison. Fisher’s exact test was used if >20% of cells had expected frequencies below five.

The Kaplan–Meier analysis and the log-rank test were used to compare outcomes of patients with VFTR and AFTR. For the analysis of predictors of outcome, patients were grouped according to the overall composite endpoint of death/rehospitalization for heart failure. Variables differing between groups were further analyzed using univariate Cox regression. Variables significant in univariate Cox regression were entered into multivariate Cox regression using a backwards conditional model. Autocorrelation and multicollinearity were investigated using Pearson’s and Spearman’s correlation coefficient as well as the variance inflation factor (VIF). A correlation of r ≥ 0.4 and VIF ≥ 10 were prespecified cut-offs for significant multicollinearity.

The results from the Cox regression were shown as hazard ratios (HR) and their respective 95% confidence interval (CI). AFTR, female gender, coronary artery disease (CAD), estimated glomerular filtration rate (eGFR) and residual TR ≤ II were entered into the multivariate model. The EuroScore II was not entered into the model for obvious autocorrelation with gender and comorbidities, which are included in that score.

SPSS Version 29 (IBM, New York, NY, USA) was used for the statistical analysis. All testing was performed two-sided, and a *p*-value of <0.05 was considered significant for all testing.

## 3. Results

### 3.1. Baseline Patient Demographics

Of 136 patients undergoing T-TEER between 2022 and 2024, 27 patients (19.9%) were classified as having AFTR. Patients with VFTR (N = 109) and AFTR (N = 27, 19.9%) were both elderly (82.0 {interquartile range: 74.5–84.5} vs. 82.0 {IQR: 75.0–84.0} years, *p* = 0.98) and had similar interventional risk according to the EuroScore II (7.1 {IQR: 4.5–12.2} vs. 4.7 {IQR: 3.4–9.7}%, *p* = 0.3) (see Table 1). However, the TRI-SCORE tended to be worse in patients with VFTR (5.0 {IQR: 3.0–6.0} vs. 4.0 {IQR: 2.0–5.0}%, *p* = 0.08). Atrial fibrillation was equally frequent in both groups (89.9 vs. 88.9%, *p* = 0.88). AFTR patients were significantly more often female (56.0 vs. 77.1%, *p* = 0.04) and had lower NT-proBNP (3600.0 {1706.0–6302.0} vs. 1988.0 {1034.8–3723.3} pg/mL, *p* < 0.01). All patients were highly symptomatic, with NYHA class ≥ III in 87.1% and 81.5% of patients (*p* = 0.68).

### 3.2. Echocardiographic Differences Between AFTR and VFTR

**Figure 1:**
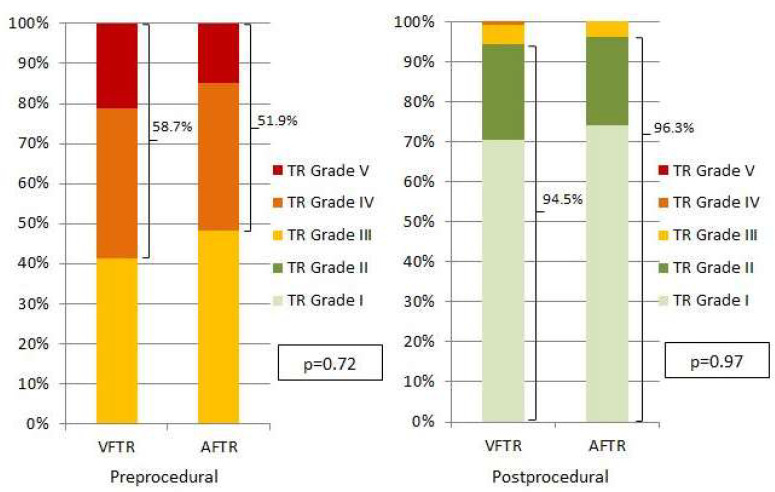
Pre- and postprocedural grades of tricuspid regurgitation. Grades of TR were equal between patients with VFTR and AFTR. In both groups, a similar reduction in TR was achieved with T-TEER.

While RVFAC (29.5 ± 8.6 vs. 42.1 ± 4.3%, *p* < 0.01) and LVEF (48.5 ± 12.3 vs. 58.6 ± 8.0%, *p* < 0.01) were expectedly lower in patients with VFTR, right atrial dilation (RA volume: 126.7 ± 56.5 vs. 127.6 ± 74.2 mL, *p* = 0.99) was similar in both patient groups (see Table 2). The grade of preprocedural TR was similar between both groups (TR ≥ IV 58.7 vs. 51.9%, *p* = 0.52, see Figure 1).

### 3.3. Procedural Details and TR Reduction

Successful T-TEER with TR reduction ≥ 2 degrees (96.3 vs. 92.6%, *p* = 0.34) was observed in both groups, and residual TR ≤ II (94.5 vs. 96.3%, *p* = 1.0) was achieved equally frequently with a comparable number of devices (≥2 devices: 67.0 vs. 55.6%, *p* = 0.27). The PASCAL Ace system was used in the majority (86.0%) of patients, while the Triclip was used in the remaining patients.

### 3.4. Incidence of the Composite Endpoint

The median time to either the composite endpoint or follow-up for the 1-year composite endpoint was 365.0 {IQR: 173.3–365.0} and 407.5 {IQR: 173.3–611.8} days for the overall (latest available follow-up) composite endpoint.

**Figure 2:**
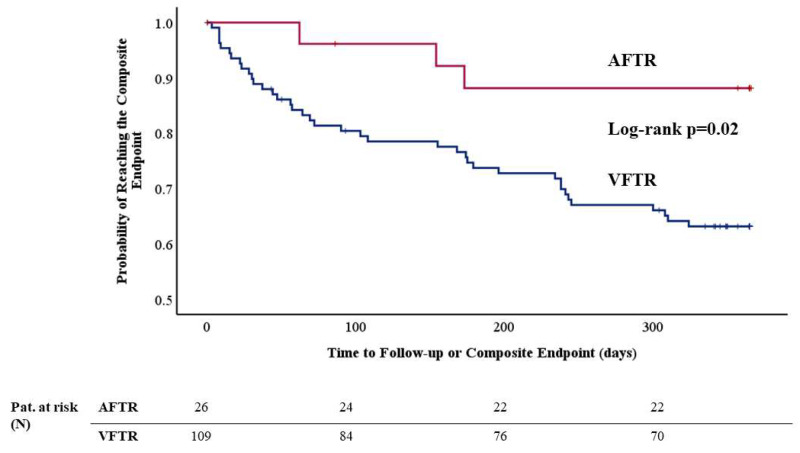
Kaplan–Meier analysis of the 1-year composite endpoint of death and rehospitalization for heart failure. The cumulative incidence of the composite endpoint occurred significantly more often in patients with VFTR compared to patients with AFTR (34.3 vs. 12.0%, log-rank *p* = 0.02).

**Figure 3:**
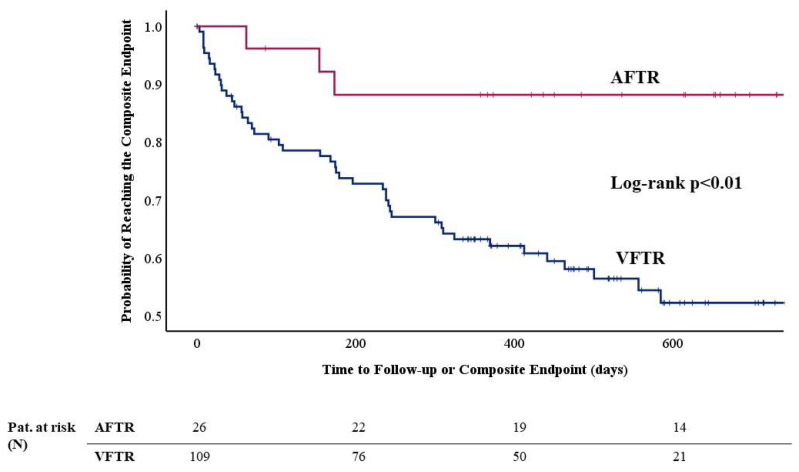
Kaplan–Meier analysis of the overall composite endpoint of death and rehospitalization for heart failure. The cumulative incidence of the composite endpoint occurred significantly more often in patients with VFTR compared to patients with AFTR (44.0 vs. 11.1%, log-rank *p* < 0.01).

The cumulative incidence of the 1-year composite endpoint was significantly higher (34.3 vs. 12.0%) in patients with VFTR (log-rank *p* = 0.02, see Figure 2). The cumulative incidence of the overall endpoint occurred significantly more often in the VFTR group as well (44.0 vs. 11.1%, log-rank *p* < 0.01, see Figure 3). The individual components of the overall composite endpoint significantly differed between AFTR and VFTR patients as well. All-cause death (32.1 vs. 11.1%, log-rank *p* = 0.01) as well as rehospitalization for decompensated heart failure occurred more often in patients with VFTR (29.4 vs. 7.4%, log-rank *p* = 0.02).

### 3.5. Predictors of the Composite Endpoint

Supplemental Tables S1 and S2 depict the baseline and echocardiographic parameters grouped according to the incidence of the overall composite endpoint. The EuroScore II (8.3 {5.3–13.0} vs. 5.0 {3.6–7.1} %, *p* < 0.01) was significantly higher in patients reaching the composite endpoint. Moreover, Nt-proBNP (3795.0 {2083.0–7837.0} vs. 2488.0 {1255.0–4865.0} pg/mL, *p* = 0.01) was significantly higher, while eGFR was lower (36.0 ± 19.9 vs. 44.9 ± 19.9 mL/min, *p* = 0.01). Patients reaching the endpoint tended to have residual TR ≤ II (90.2 vs. 97.6%, *p* = 0.06) less often. The predictors of the composite endpoint identified through univariate und multivariate Cox regression are shown in Table 3. AFTR (HR: 0.21, 95%CI: 0.06–0.7, *p* < 0.01) as well as residual TR ≤ II (HR: 0.3, 95%CI: 0.1–0.84, *p* = 0.02) were independently associated with a lower incidence of the composite endpoint in the multivariate Cox regression analysis.

## 4. Discussion

We conducted a monocenter, retrospective study comparing outcomes between patients with VFTR and AFTR after T-TEER using a novel definition of AFTR based on recently published recommendations [7]. This is the first study to compare outcomes after T-TEER using this novel definition, and the main findings can be summarized as follows (see also the Graphical Abstract):

(1) AFTR and VFTR patients seem comparable regarding age and comorbidities, although patients with AFTR are more often female (77.1 vs. 56.0%, *p* = 0.04).

(2) T-TEER seems to yield similar results in patients with AFTR and VFTR, and acceptable results may be achieved in the majority of patients (residual TR ≤ II in 94.5 vs. 96.3%, *p* = 1.0).

(3) Patients with AFTR presented with a significantly lower cumulative incidence of the 1-year composite endpoint of death/rehospitalization for heart failure (34.3 vs. 12.0%, log-rank *p* = 0.02, see Figure 2) and furthermore with a lower cumulative incidence of the overall endpoint (44.0 vs. 11.1%, log-rank *p* < 0.01, see Figure 3).

(4) AFTR (HR: 0.21, 95%CI: 0.06–0.7, *p* < 0.01) as well as residual TR ≤ II (HR: 0.3, 95%CI: 0.1–0.84, *p* = 0.02) were independently associated with a lower incidence of the composite endpoint.

Since the entity of AFTR is relatively new, lacking a guideline-based definition, little is known about the outcome of these patients after T-TEER. Russo et al. defined AFTR as TR in the context of normal left ventricular function > 50%, the presence of atrial fibrillation and sPAP < 50 mmHg and included 298 patients of the TriValve Registry [11]. Of all included, 22% of patients were classified as having AFTR, who had better survival after TEER compared to patients with VFTR after a median follow-up of 10 months (*p* = 0.03). In multivariate Cox regression, VFTR showed a strong tendency as a predictor of mortality but missed statistical significance (HR 2.94, 95%CI: 0.97–8.94, *p* = 0.057) [11]. Russo et al. used the definition of “isolated TR” outlined in the ACC/AHA guidelines as a surrogate for AFTR [11,17], which is the first official guideline describing this type of “isolated TR” with association to atrial fibrillation, LVEF > 60%, sPAP < 50 mmHg and absence of left-sided heart disease [17]. Thus, the guideline highlights the growing interest in isolated TR due to the rising number of patients with right-sided heart failure stemming from isolated TR [17]. The definition of Schlotter et al. included several echocardiographic parameters (TV tenting height, RV diameters and LVEF ≥ 50%) identified through a cluster analysis, not including parameters for RA/RV function or sPAP [8]. Galloo et al. analyzed outcomes of 1037 patients with severe TR receiving conservative treatment [9]. Similar to the definition used in our study, Galloo et al. used RV function measured by RVFAC and/or TAPSE, sPAP and LVEF for the definition of AFTR [9]. Recently published data from the multicenter EURO-TR registry used preserved RV function measured as TAPSE > 17 mm and the RA/RV area ratio (≥1.5) to define AFTR, not taking into account LV function or sPAP [12]. This definition classified 30% of patients in their cohort as having AFTR [12], a higher proportion compared to previous studies [8,9,11].

Despite differing definitions of AFTR (see also Table S3), it is commonly defined by the absence of RV and LV dysfunction and thus is a rule-out diagnosis. In AFTR, regurgitation is mainly caused by a dilatation of the tricuspid annulus caused by an imbalance of leaflet and annulus due to RA dilatation and dysfunction [7]. In contrast to the mitral valve apparatus, which is more strongly stabilized by the papillary muscles, the papillary muscles supporting the tricuspid valve are smaller and more separated and the tricuspid annulus has less fibrotic tissue, both factors favoring tricuspid annular dilatation [7]. Therefore, AFTR presents the Carpentier Type I with structural normal leaflets and normal motion with isolated annular dilatation [7]. In VFTR, RV dysfunction and enlargement causes basal ventricular dilatation, an ellipsoidal-shaped ventricular remodeling, resulting in displacement, tethering and tenting of the papillary muscles [5]. Differentiation of these etiologies may be challenging, especially due to overlapping characteristics in advanced stages. Most studies use LVEF [8,9,11] to account for LV dysfunction, whereas there seems to be less uniformity regarding RV function. Notably, atrial fibrillation is not required for a diagnosis of AFTR according to the recent expert recommendations [7], as AFTR may occur even in the absence of atrial fibrillation. Contrarily, these recommendations [7] describe atrial fibrillation as an overlapping feature seen both in VFTR and AFTR [7]. Thus, we did not include atrial fibrillation in our classification of AFTR, in contrast to Russo et al. [11]. Furthermore, we could not find a difference in the prevalence of atrial fibrillation in patients with AFTR and VFTR (89.8% and 88.9%, *p* = 0.88).

In the present study, we used RVFAC ≥ 35% as an easy-to-measure parameter of normal RV function and excluded left ventricular impairment (LVEF ≤ 50%) and RV dysfunction through severe pulmonary hypertension (≥50 mmHg) to define AFTR. Nevertheless, 19.9% of patients were defined as having AFTR with our classification, a proportion similar to previous studies [8,9,11]. Moreover, our population showed similar patient characteristics when compared to former studies investigating AFTR: a greater proportion of female patients (77 vs. 56.0%, *p* = 0.04) and lower NT-proBNP [8,9,11].

AFTR conferred a better prognosis with regard to the composite endpoint compared to VFTR (HR: 0.21, 95%CI: 0.06–0.7, *p* < 0.01) despite a similar TR reduction through T-TEER in our study. This is in line with the results from Schlotter et al., who also identified AFTR as an independent predictor for mortality and heart failure rehospitalization [8]. Although Russo et al. used a slightly different definition of AFTR, they concluded that AFTR was associated with better survival after T-TEER in their study [11]. Furthermore, data from the EURO-TR registry observed better outcomes in patients with AFTR in the 2-year follow-up [12]. In contrast to our study, they observed better procedural TR reduction in patients with AFTR as well [12].

However, T-TEER is a safe and efficient procedure, resulting in a sustained reduction in TR [18,19] and an improvement in functional status and quality of life [20]. Moreover, the degree of TR reduction and residual TR predicted outcomes after T-TEER in previous, observational studies [21,22,23]. Three-year follow-up data from the randomized controlled TRILUMINATE trial confirmed that patients with TR reduction to moderate or less TR had a better prognosis compared to patients who had greater-than-moderate residual TR [24]. In our study, residual TR ≤II was also identified as an independent predictor of the composite endpoint of death or heart failure rehospitalization (HR 0.3, 95%CI: 0.1–0.84, *p* = 0.02) in multivariate Cox regression. This further emphasizes the need to aim for optimal procedural results, as this affects patients’ prognoses.

Unfortunately, data regarding the outcomes of patients grouped according to FTR etiology from the TRILUMINATE trial are lacking so far.

TR is a condition associated with poor prognosis [3,4,5,25,26], but more data are necessary to identify those patients who can benefit the most from TEER. Due to the isolated pathology of the right atrium, the tricuspid annulus and the lack of severe right or left ventricular heart disease as well as severe pulmonary hypertension, the targeted treatment of TR may simply exert a greater health benefit and confer a better prognosis in patients with AFTR. Contrarily, VFTR is associated with remodeling of the right ventricle, which negatively affects the tricuspid valve apparatus as well as the right atrium. These changes might be less amenable to T-TEER.

While T-TEER was not compared to medical therapy alone in our present study, the better outcome of patients with AFTR suggests that these patients are especially suitable candidates for T-TEER. Nevertheless, our data do not suggest or support withholding T-TEER from patients with VFTR but solely emphasize a potentially greater benefit in patients with AFTR.

Furthermore, data from randomized controlled trials comparing medical therapy and T-TEER in patients with AFTR would be desirable, as there is a lack of data on optimal management strategies of patients with AFTR. At last, future studies analyzing long-term outcomes comparing AFTR and VFTR are warranted. Considering the growing evidence for better outcomes in AFTR patients, it remains to be seen if future guidelines will address the differences of AFTR and VFTR patients. Although it must be considered speculative at this point, AFTR could receive a stronger recommendation for T-TEER treatment in future guidelines.

### Limitations

This study is a retrospective observational study with a limited sample size from a single center with all limitations inherent to such a study. Moreover, local practice and expertise may affect the results and limit the generalizability of the findings. We presented data from an all-comer registry with consecutive patients to minimize selection bias. Multivariate Cox regression was used to study independent predictors of outcome and to minimize confounding bias; however, confounding can ultimately not be ruled out in a retrospective study. Definitions of AFTR vary across several studies and limit the comparability of results. Most notably, we did not require the presence of AF in patients with AFTR. However, we used a definition with easily obtainable and practicable parameters to define AFTR according to a recently published expert recommendation by members of the TVARC [7,14]. CIED-induced TR is considered a distinct entity different from FTR, and such patients were excluded from this analysis; any findings from this study may not be applied to patients with CIED-induced TR.

RV and LV function were assessed using two-dimensional parameters, while the use of 3D TTE (3D RVEF or 3D LVEF) or speckle-tracking analysis (LV/RV strain) may enhance the detection of ventricular dysfunction, and such techniques may further improve the distinction of AFTR and VFTR. Our study solely focused on the definition of AFTR and VFTR and the outcome of these patient groups. Procedural details, e.g., clip position, procedural duration or the patients’ medication, were not analyzed. Further studies, especially multicenter studies, are necessary to analyze and compare treatment options in patients with AFTR and VFTR.

## 5. Conclusions

Our study confirms that TEER can be performed in patients with AFTR and VFTR with similar procedural results. Despite equally effective TR reduction through T-TEER in AFTR and VFTR, AFTR was significantly associated with a lower incidence of the composite endpoint of rehospitalization for decompensated heart failure and all-cause death.

## Figures and Tables

**Table 1:** Baseline patient characteristics.

	Total (N = 136)	VFTR (N = 109)	AFTR (N = 27)	*p*-Value
Age, years	82.0 {75.0–84.0}	82.0 {74.5–84.5}	82.0 {75.0–84.0}	0.98
BMI, kg/m^2^	25.3 ± 4.8	25.7 ± 4.9	24.1 ± 4.3	0.13
Female, N (%)	82 (60.3)	61 (56.0)	21 (77.8)	**0.04**
AHT, N (%)	117 (86.0)	95 (87.2)	22 (81.5)	0.53
Diabetes mellitus, N (%)	33 (24.3)	27 (24.8)	6 (24.3)	0.78
CAD, N (%)	77 (56.6)	61 (56.0)	16 (56.3)	0.76
AF, N (%)	122 (89.7)	98 (89.9)	24 (88.9)	0.88
Permanent AF, N (%)	46 (33.8)	36 (33.0)	10 (37.0)	0.69
PM/ICD/CRT, N (%)	38 (27.9)	31 (28.4)	7 (25.9)	0.79
NYHA II, N (%)	19 (14.0)	14 (12.8)	20 (74.1)	0.68
NYHA III, N (%)	103 (75.7)	83 (76.1)	20 (74.1)	
NYHA IV, N (%)	14 (10.3)	12 (11.0)	2 (7.4)	
Euro SCORE II, %	5.9 {4.0–9.6}	6.1 {4.0–9.8}	4.7 {3.6–9.6}	0.3
TRI-SCORE, %	4.0 {3.0–6.0}	5.0 {3.0–6.0}	4.0 {2.0–5.0}	0.08
NT-proBNP, pg/mL	2879.5 {1506.0–6285.8}	3600 {1706.0–6302.0}	1988.0 {1034.8–3723.3}	**<0.01**
eGFR, mL/min	41.6 ± 20.3	40.1 ± 20.8	47.9 ± 17.3	0.074

Values are shown as frequencies (N) and percentages (%), mean ± standard deviation (SD) or median and IQR in parentheses. Abbreviations: AF = atrial fibrillation; CAD = coronary artery disease; CRT = Cardiac Resynchronization Therapy; eGFR = estimated glomerular filtration rate; ICD = implantable cardioverter defibrillator; FTR = functional TR; NT-proBNP = N-terminal pro hormone brain natriuretic peptide; NYHA = New York Heart Association; PM = pacemaker; TR = tricuspid regurgitation. Bold numbers indicate significant *p*-values.

**Table 2:** Echocardiography and procedural results.

	Total (N = 136)	VFTR (N = 109)	AFTR (N = 27)	*p*-Value
LVEF, %	50.6 ± 12.2	48.5 ± 12.3	58.6 ± 8.0	**<0.01**
RA Volume, mL	127.6 ± 60.9	127.6 ± 56.5	127.6 ± 74.2	0.99
RV FAC, %	32.0 ± 9.4	29.5 ± 8.6	42.1 ± 4.3	**<0.01**
TAPSE, mm	18.4 ± 5.1	17.9 ± 5.0	20.6 ± 5.2	0.053
sPAP, mmHg	50.3 ± 13.0	52.7 ± 13.1	41.1 ± 7.5	**<0.01**
EROA, cm^2^	0.58 ± 0.2	0.57 ± 0.2	0.62 ± 0.3	0.38
VC biplane, mm	10.2 ± 3.7	10.0 ± 3.3	10.7 ± 4.8	0.52
Grade of TR pre				
III	58 (42.6)	45 (41.3)	13 (48.1)	0.72
IV	51 (37.5)	41 (37.6)	10 (37.0)	
V	27 (19.9)	23 (21.1)	4 (14.8)	
Grade of TR pre ≥ IV, N (%)	78 (57.4)	64 (58.7)	14 (51.9)	0.52
Grade of TR post				
IV	1	1 (0.9)	0	0.97
III	6 (4.4)	5 (4.6)	1 (3.7)	
II	32 (22.9)	26 (23.9)	6 (22.2)	
≤I	97 (71.5)	77 (70.6)	20 (74.1)	
TR Grade Reduction ≥ 2 degrees, N (%)	130 (95.6)	105 (96.3)	25 (92.6)	0.34
Residual TR ≤ II, N (%)	129 (94.9)	103 (94.5)	26 (96.3)	1.0
≥2 Devices, N (%)	88 (64.7)	73 (67.0)	15 (55.6)	0.27

Values are shown as frequencies (N) and percentages (%), mean ± standard deviation (SD) or median and IQR in parentheses. Abbreviations: EROA = effective regurgitant orifice area, LVEF = left ventricular ejection fraction, pre = preprocedural (baseline), RA = right atrium, RV = right ventricle, sPAP = systolic pulmonary artery pressure, TAPSE = tricuspid annular plane systolic excursion, TR = tricuspid regurgitation, VC = vena contracta. Bold numbers indicate significant *p*-values.

**Table 3:** Cox regression for predictors associated with the composite endpoint of death/rehospitalization for decompensated heart failure.

	Univariate			Multivariate		
Parameter	HR	95%CI	*p*	HR	95%CI	*p*-Value
AFTR	0.18	0.056–0.6	**<0.01**	0.21	0.06–0.7	**<0.01**
Female	0.47	0.27–0.81	**<0.01**	0.59	0.33–1.05	0.07
CAD	2.3	1.27–4.3	**<0.01**	2.6	1.34–5.0	**<0.01**
NT-proBNP, pg/mL	1.02	0.99–1.1	0.08			
eGFR, mL/min	0.98	0.96–0.99	**<0.01**	0.98	0.96–0.99	**0.03**
RA Volume, mL	1.0	0.99–1.01	0.14			
Residual TR ≤ II	0.14	0.14–0.88	**0.03**	0.3	0.1–0.84	**0.02**

The variables are shown as hazard ratios (HR) and their respective 95% confidence interval (CI). The left side of the table shows the results from the univariate Cox regression analysis. The variables significant in univariate Cox regression were entered into a multivariate Cox regression model using a backwards conditional model. Abbreviations: AFTR = atrial functional tricuspid regurgitation, CAD = coronary artery disease, eGFR = estimated glomerular filtration rate, NT-proBNP = N-terminal pro hormone brain natriuretic peptide, RA = right atrial, TR = tricuspid regurgitation. Bold numbers indicate significant *p*-values.

## Data Availability

The original contributions presented in this study are included in the article/Supplementary Material. Further inquiries can be directed to the corresponding author.

## Supplementary Materials

The following supporting information can be downloaded at: https://www.mdpi.com/article/10.3390/jcm14030794/s1. Table S1. Comparison of patients with/without the composite endpoint (death or rehospitalization due to decompensated heart failure); Table S2. Baseline echocardiography and procedural results of patients with/without the composite endpoint (death or rehospitalization due to decompensated heart failure); Table S3. Overview regarding different definitions of AFTR across studies.
