# Supplementary material for: Atrial Functional Tricuspid Regurgitation (AFTR) Is Associated with Better Outcome After Tricuspid Transcatheter Edge-to-Edge Repair (T-TEER) Compared to Ventricular FTR (VFTR)
Source: J Clin Med. 2025 Jan 25;14(3):794. doi: 10.3390/jcm14030794 (PMC11818297; doi:10.3390/jcm14030794)
Supplement: Supplementary file 1 [file jcm-14-00794-s001.zip › jcm-3419182-supplementary.pdf]

Journal of Clinical Medicine

Supplementary Materials

Table S1. Comparison of patients with/without the composite endpoint (death or rehospitalization due to decompensated heart failure).

Total<br>N = 136	Total<br>N = 136	Endpoint Not Reached<br>(N = 85)	Endpoint Reached<br>(N = 51)	*p*
AFTR, N (%)	27 (19.9)	24 (28.2)	3 (5.9)	**<0.01**
Age, years	82.0 {75.0–84.0}	82.0 {75.0–84.0}	82.0 {75.0–85.0}	0.62
Female, N (%)	82 (60.3)	59 (69.4)	23 (45.1)	**0.01**
AHT, N (%)	117 (86.0)	73 (85.9)	44 (86.3)	0.95
Diabetes mellitus, N (%)	33 (24.3)	17 (20.0)	16 (31.4)	0.13
CAD, N (%)	77 (56.6)	41 (48.2)	36 (70.6)	**0.01**
AF, N (%)	122 (89.7)	77 (90.6)	45 (88.2)	0.66
Permanent AF, N (%)	46 (33.8)	30 (35.3)	16 (31.4)	0.64
PM/ICD/CRT, N (%)	38 (27.9)	20 (23.5)	18 (35.3)	0.14
NYHA II, N (%)	19 (14.0)	12 (14.1)	7 (13.7)	
NYHA III, N (%)	103 (75.7)	68 (80.0)	35 (68.6)	0.89
NYHA IV, N (%)	14 (10.3)	5 (5.9)	9 (17.6)	
Euro SCORE II, %	5.9 {4.0–9.6}	5.0 {3.6–7.1}	8.3 {5.3–13.0}	**<0.01**
TRI-SCORE, %	4.0 {3.0–6.0}	4.0 {2.0–5.0}	6.0 {4.0–7.0}	**<0.01**
NT-proBNP, pg/mL	2879.5 {1506.5–6285.8}	2488.0 {1255.0–4865.0}	3795.0 {2083.0–7837.0}	**0.01**
eGFR, ml/min	41.6 ± 20.3	44.9 ± 19.9	36.0 ± 19.9	**0.01**

Values are shown as frequencies (N) and percentages (%), mean ± standard deviation (SD) or median and IQR in parentheses. Abbreviations: AF = atrial fibrillation; CAD = coronary artery disease; CRT = Cardiac Resynchronization Therapy; eGFR = estimated glomerular filtration rate; FTR = functional TR; NT-proBNP = N-terminal pro hormone brain natriuretic peptide; NYHA = New York Heart Association; STS = Society of Thoracic Surgeons; TR = tricuspid regurgitation. Bold numbers indicate significant *p*-values.

Table S2. Baseline echocardiography and procedural results of patients with/without the composite endpoint (death or rehospitalization due to decompensated heart failure).

	Total<br>N = 136	Endpoint Not Reached<br>(N = 85)	Endpoint Reached<br>(N = 51)	*p*
LVEF (%)	50.6 ± 12.2	52.9 ± 11.9	46.6 ± 11.7	**0.03**
RA volume (mL)	127.6 ± 60.9	121.8 ± 60.9	137.3 ± 60.2	**0.02**
RV FAC (%)	32.0 ± 9.4	33.5 ± 8.3	29.5 ± 10.5	**0.01**
TAPSE (mm)	18.4 ± 5.1	19.0 ± 5.4	17.3 ± 4.4	0.1
sPAP (mmHg)	50.3 ± 13.0	47.5 ± 13.1	55.2 ± 11.4	**<0.01**
EROA (cm^2)	0.6 ± 0.2	0.6 ± 0.2	0.6 ± 0.3	0.39
VC biplane (mm)	10.2 ± 3.7	10.2 ± 3.8	10.1 ± 3.5	0.86
TR volume (mL)	46.8 ± 18.3	44.4 ± 18.1	51.1 ± 18.1	0.06
Grade of TR pre				
III	58 (42.6)	39 (45.9)	19 (37.3)	
IV	51 (37.5)	32 (37.6)	19 (37.3)	0.39
V	27 (19.9)	14 (16.5%)	13 (25.5)	
≥2 Devices (%)	88 (64.7)	58 (68.2)	30 (58.8)	0.27
Grade of TR post				

IV	1 (0.7)	0	1 (2.0)	0.051
III	6 (4.4)	2 (2.4)	4 (7.8)	
II	32 (23.5)	16 (18.8)	16 (31.4)	
≤I	97 (71.3)	67 (78.8)	30 (58.8)	
TR grade reduction ≥ 2 degrees	130 (95.6)	83 (97.6)	47 (92.2)	0.13
Residual TR ≤ II	129 (94.9)	83 (97.6)	46 (90.2)	0.06

Values are shown as frequencies (N) and percentages (%), mean ± standard deviation (SD) or median and IQR in parentheses. Abbreviations: EROA = effective regurgitant orifice area, IVC = inferior vena cava, LVEF = left ventricular ejection fraction, pre = preprocedural (baseline), RA = right atrium, RV = right ventricle, sPAP = systolic pulmonary artery pressure, TAPSE = tricuspid annular plane systolic excursion, TR = tricuspid regurgitation. Bold numbers indicate significant *p*-values.

Table S3. Overview regarding different definitions of AFTR across studies.

**First Author, Year**	**Definition of AFTR**
Otto C., 2020 (ACC/AHA Guidelines) [17]	"Isolated TR" in association with the following:<br>- Presence of atrial fibrillation;<br>- LVEF ≥ 60%;<br>- sPAP < 50 mmHg;<br>- Exclusion of left-sided heart disease;<br>- Normal appearance of tricuspid leaflets.
Schlotter F., 2022 [8]	- Tenting height ≤ 10 mm<br>- RV midventricular diameter ≤ 38 mm<br>- LVEF ≥ 50%
Gavazzoni M., 2022 [10]	- Presence of atrial fibrillation<br>- LVEF ≥ 60%<br>- sPAP < 50 mmHg<br>- No left-sided heart valvular disease<br>- Normal appearance of tricuspid leaflets
Russo G., 2023 [11]	- LVEF > 50% without wall abnormalities<br>- Presence of atrial fibrillation<br>- sPAP < 50 mmHg<br>- Exclusion of primary TR and CIED
Galloo X., 2023 [9]	- Exclusion of left-sided heart disease (LVEF ≥ 50%) and/or valvular disease (mitral regurgitation or aortic stenosis)<br>- sPAP < 50 mmHg<br>- RVFAC ≥ 30% and/or TAPSE ≥ 15 mm<br>- No other cause than RA dilatation
Stolz L., 2024 [12]	- Ratio of end-systolic RA area/RV area ≥ 1.5 in the presence of preserved RV function with TAPSE > 17 mm
Muraru D., 2024 [7]	AFTR requires fulfilment of all six criteria; probable AFTR by at least 4 criteria:<br>- Clinically relevant secondary TR;<br>- Predominant TA dilatation;<br>- Predominant RA dilatation with increased end-systolic RA/RV ratio;<br>- Absence of significant leaflet tethering;<br>- RV conical remodeling with predominant enlargement of RV basal dimension;<br>- Preserved LV and RV systolic function.
